# Assessing patterns of eavesdropper risk on sexual signals and the use of meta-analysis in behavioural ecology: a comment on: ‘The exploitation of sexual signals by predators: a meta-analysis' White *et al*. (2022)

**DOI:** 10.1098/rspb.2022.1866

**Published:** 2023-05-10

**Authors:** Ximena E. Bernal, Brian C. Leavell, Rachel A. Page

**Affiliations:** ^1^ Department of Biological Sciences, Purdue University, 915 W State Street, West Lafayette, IN 47907, USA; ^2^ Smithsonian Tropical Research Institute, Apartado 0843-03092, Balboa, Ancón, Republic of Panamá

In their recent meta-analysis, White *et al*. [1] cover a wide range of literature, drawing on eavesdropping studies that span much of the animal world, to examine how eavesdropping predators impose selection pressure on sexual signallers. This is an interesting phenomenon that has received previous attention but has not been investigated using a quantitative approach [2–4]. We applaud their extensive investigation into this fascinating literature. We disagree, however, with the claim that ‘contexts in which sexual signalling may incur no cost, or even reduce the incidence of predation, are common’. We argue that in attempting to identify generalities within and across methodological paradigms and sensory modalities, the methodology used may not sufficiently account for the complex causal forces underlying the phenomenon at hand. We find that the increasingly popular meta-analysis, an effective tool to evaluate support for various hypotheses, can miss subtleties of the field and the biology of the phenomenon investigated. Like any model, it is as good as its inputs—a rule of thumb that has been previously illustrated in other modelling contexts [5]. We highlight how, while models can encompass broad biological diversity, nuances among studies may reveal diverse strategies rather than statistical noise. Eavesdropping of mating signals, in particular, is a behaviour that occurs across disparate sensory modalities, taxonomic groups and ecological contexts. A variety of factors, for example, have been identified to modulate the risk of signallers to attacks by eavesdropping enemies [3,6,7]. In addition, factors other than signal exploitation can ultimately drive signal evolution (e.g. [8]). Even though this field is starting to thoroughly understand the complex drivers and consequences of signal exploitation, such as the direct and indirect effects that eavesdroppers may have on non-signaller parties [9–12], investigations of eavesdropping on mating signals benefit from considering the complexity of this phenomenon.

The main claim of the meta-analysis is based on the assumption that eavesdropping risk is modality-specific, driven by the physical properties of the sensory modality of the broadcast signal. While vulnerability to signal exploitation may be expected to vary by signal sensory modality, the underlying assumption that the active space of a signal is generalizable by its physical characteristics can be questioned. The generalized predictions presented in White *et al*.'s study may be misleading, as the magnitude of eavesdropping risk is context-dependent and influenced by various factors including signalling environments, the eavesdropper's sensory system and prey signalling strategies (reviewed in [4]). For instance, one prediction in this review is that visual signals incur low eavesdropper risk, as they propagate in the short/medium range. Yet, rather than absolute transmission distance, signal vulnerability depends on the scale at which eavesdroppers and signallers interact. Considering the ecology of these interactions probably increases their risk factor, which is also heightened by visual signals being more accurately localized than signals in many other sensory modalities. In addition to varying in the space over which they may be detected, visual signals also vary in their temporal availability to potential exploiters. Some visual signals are continuously displayed, unceasingly exposing the signaller to potential eavesdroppers (e.g. [13,14]). By contrast, other visual signals can be selectively turned ‘*off*’ and ‘*on*' by exposing colour patches (e.g. [15]) or by engaging in specific body postures and movements (e.g. [16]), modulating the signaller's risk to eavesdroppers. The nuances of these signalling strategies within their ecological contexts are thus key to unlocking their realized costs in the wild.

In an analogous way, the prediction that acoustic signals always have long ranges is also a broad generalization, as acknowledged in the study's discussion. High-frequency and low-intensity acoustic signals, for example, attenuate greatly over short distances and are presumably deployed to avoid detection by eavesdropping enemies [17–21]. Moreover, assessments of the range of signals that ignore the spatial scale at which animals interact can provide a narrow view of a signaller's risk. Even if a signal has a restricted range, it can still expose the signaller to a large community of exploiters. Vibrational signals, for example, are detectable only at very close range and were thus assumed to be safe from exploitation by eavesdropping enemies [22]. Recent investigations, however, have shown that the opposite is true. While substrate-borne vibrations do not transmit far, they are one of the most taxonomically widespread forms of communication, as sensory receptors for their detection are nearly ubiquitous across species [23,24]. Eavesdropper risk of vibrational signals is thus much higher than historically assumed [25], and this sensory modality provides an example of how generalizations focused on signal range can bias our understanding of signaller vulnerability.

White *et al*. point out that most studies included in their meta-analysis overlook the influence of behaviour by signallers and eavesdroppers. They also caution that understanding the influence of behaviour on risk from eavesdropping enemies ‘demands deeper knowledge of the structure and diversity of sensory environments, signals, and receivers’ [1]. To their latter point, we fully agree. For instance, several studies incorporated in the predation risk analysis used stationary clay models in the wild, a method with well-known limitations due to the importance of prey movement to certain predators [26,27]. Indeed, in the meta-analysis, nine of the 10 studies reporting negative predation risk on conspicuous visual signals used models. We question the conclusion that visual signals incur little predation risk and posit that this interpretation may have been driven by studies that did not consider the signaller's behaviours.

While we agree with White *et al*. that a deeper investigation into the behaviour of signaller and eavesdropper should be a key goal in eavesdropping studies going forward, it is worth highlighting that some studies have taken large steps in that direction. Indeed, many rigorous studies examining eavesdropping on sexual signals demonstrate the value of this approach but were not included in this study's analysis (e.g. [13,28–34]). We therefore argue that accounting for and parsing the interactions of multiple causal drivers (including behaviour) on the magnitude of eavesdropper risk on sexual signals is critical to moving forward towards identifying generalities.

More broadly, we challenge the conclusion that in the wild there commonly is no risk from eavesdroppers. We suspect this result reflects the nature of their dataset rather than a biological generality. Specifically, their selection criteria excluded all studies in which comparators received zero attacks from eavesdropping enemies, reducing their total number of records by almost half (excluding 101 of 216 total records). These exclusions probably led to an underestimation of the predation risk on signalling individuals. Similarly, the selection criteria used to include studies may bias the outcome. One study included in this meta-analysis, for example, revealed that a conspicuous signal incurred reduced predation risk compared with cryptic morphs [35]. The conspicuous signal, however, was novel to the predators on that continent and, therefore, processes such as neophobia could overshadow the effect of conspicuousness on predation risk. This experiment thus tested the influence of novelty on predation risk, not predation risk of an ecologically relevant signal in the wild. Such effects may thus not represent reduced overall predation risk. The selection criteria and assumptions of the meta-analysis may have biased the conclusion and further work is necessary to re-examine whether eavesdropping risk is commonly absent in the wild.

In examining eavesdropping risk, White *et al*. focused on studies that address enemies exploiting communication systems, not including studies reporting anti-eavesdropper strategies deployed by signallers. When considering current signal features and signalling displays, evidence suggests intense past predation pressures have driven the evolution of a broad variety of signal features and signalling behaviours that conceal the signaller (reviewed in [4]). Strategies that reduce signal detection (e.g. [36–38]), localization (e.g. [39,40]) and attractiveness (e.g. [41]) are widespread across taxa and sensory modalities. Eavesdroppers are also frequently associated with curtailing signal ornamentation (e.g. [6]) and, in extreme but limited cases, driving signal loss (e.g. [42]) and re-evolution of novel, safer signals (e.g. [43,44]). White *et al*. acknowledge eavesdropper-induced shifts in signalling behaviour are well documented, but including this evidence in their analysis and interpretations could inform the conclusions more broadly. Ultimately, the ubiquitous nature of anti-eavesdropper strategies questions the claim that contexts in which sexual signalling may incur no eavesdropper cost are common.

Another aim of the study was to evaluate whether differences in signal salience modulate eavesdropper risk. This question is central to the theory of exploitation of sexual signals, which often assumes that conspicuousness mediates the risk faced by signallers. The conclusion was that signal salience, estimated as the degree of signal variation in the manipulations, does not affect eavesdropper risk. Here, we question the approach of assigning studies into ‘discrete’ and ‘continuous’ manipulation categories to assess signal salience. Studies using a control stimulus in which the signal was absent were categorized as ‘discrete manipulation’, with the signal interpreted as being highly conspicuous (as compared with the control). By contrast, when eavesdroppers were experimentally exposed to graded variation among the stimuli offered, studies were categorized as ‘continuous manipulation’, with signals interpreted as having lower conspicuousness. While these methodological paradigms deserve different consideration, rather than assessing signal salience, these approaches assess different perceptual and cognitive challenges for the receiver. When organisms are presented with a single stimulus (discrete manipulation), we learn about whether this signal evokes (or not) a response from a potential non-target receiver. Such an approach thus allows us to examine whether a signal is detected, recognized and ultimately elicits a given behaviour (e.g. approach and attack) in the receiver thus confirming that a given predator, parasite or parasitoid is eavesdropping on the signal. By contrast, eavesdroppers presented with two forms of a signal (continuum manipulation) can express a bias for one variant over the other. These experiments thus provide a perspective on the relative eavesdropping costs associated with signals produced in different contexts or by different individuals. The contrast between the findings of studies using those two experimental approaches, however, does not address differences in signal salience. The analysis provided thus does not directly evaluate the prediction ‘that predation risk should be heightened among discretely manipulated stimuli owing to the increased conspicuousness and salience of signallers relative to controls'. The findings of no differences in risk of exploitation among manipulations could lead to the conclusion that there is a lack of support for the assumption that increased signal salience increases signal exploitation risk. This relationship, however, is still missing direct examination integrating studies across species and sensory modalities.

The argument for the lack of evidence of signal saliency driving eavesdropper risk may also be problematic given the vague use of the terms ‘conspicuousness' and ‘saliency’ in the literature [4]. Conspicuousness and saliency are used as broad concepts that involve differences in signal detectability, localizability or preference by a receiver tapping into a variety of perceptual and cognitive processes that result in increased attractiveness. These terms are also often used based on signal conspicuousness as perceived by the researcher or assigned *a posteriori* based on the outcome of behavioural experiments. Not accounting for the sensory ecology of the receiver when identifying signal conspicuousness can be particularly problematic considering the diverse perceptual and cognitive systems of target and non-target receivers in communication networks. Such different receivers can perceive and thus respond in divergent ways to the same signal or signalling display (e.g. [45,46]). Receiver-specific considerations for each are thus probably necessary when evaluating signal salience.

We offer a final thought about the implementations of meta-analyses. We recognize and value the worthwhile enterprise of obtaining a general overview of the current landscape of knowledge in a given field to identify overarching patterns. The use of this quantitative approach can be a powerful tool but may not be appropriate to evaluate all phenomena [47,48]. In particular, challenges arise when applying meta-analysis-based generalizations to complex phenomena such as behaviour. The risk imposed by eavesdropping enemies on sexual signals, for example, is highly context-dependent, and shifts with diverse and dynamically changing ecological factors. Challenges in identifying overarching patterns are not unique to behavioural ecology; ecological interactions have long been recognized as notoriously difficult to generalize. As Lawton [49] pointed out over two decades ago, ‘community ecology is a mess, with so much contingency that useful generalisations are hard to find’. Concerns about ecological generalizations including the role of quantitative approaches [50] and simplification [51] remain as current today as they were two decades ago. As scientists, we are committed to searching for patterns and better understanding underlying commonalities. In pursuit of these worthy goals, however, care to avoid oversimplification is necessary to prevent us from inadvertently missing the stunning complexity before us.

## Data Availability

This article has no additional data.
